# Study on the impact of digital economy on industrial collaborative agglomeration: Evidence from manufacturing and productive service industries

**DOI:** 10.1371/journal.pone.0308361

**Published:** 2024-08-08

**Authors:** Hongbo Lei, Caihong Tang, Yunfei Long

**Affiliations:** 1 School of Finance, Southwestern University of Finance and Economics, Wenjiang District, Chengdu, Sichuan, China; 2 School of Economics and Management, Panzhihua University, Panzhihua, Sichuan, China; Sichuan Agricultural University, CHINA

## Abstract

In the digital era, digital economy has a far-reaching impact on the collaborative agglomeration of manufacturing and service industries. This research aims to examine the economic relationship between digital economy and industrial collaborative agglomeration. Based on a panel data set of 286 Chinese cities, this research employs Tobit model, moderating effect model, and mediating effect model to conduct data analysis. It is found that digital economy has a nonlinear relationship with industrial collaborative agglomeration, and this relationship is a U-shape. Moderating effect analysis reveals that government intervention significantly regulates the role of digital economy in industrial collaborative agglomeration. Mediating effect analysis indicates that digital economy promotes industrial collaborative agglomeration through entrepreneurial activity. Heterogeneity analysis shows that the facilitating effect of digital economy on collaborative agglomeration in high-end industries comes earlier than in middle- and low-end industries. Moreover, this research finds that digital economy plays a significant role in industrial collaborative agglomeration in central and western regions of China but not in the eastern region. To enhance the impact of digital economy on industrial collaborative agglomeration, it is crucial to strengthen the engagement of the government and ensure the availability of digital technology.

## 1. Introduction

China’s economy is transforming from a high-speed growth stage to a high-quality development stage. It is also optimizing its economic structure and changing its mode of economic development. With the transformation of China’s economic development mode, industrial collaborative agglomeration is becoming more and more obvious [1]. As an intermediate industry of input, productive service industry runs through the whole industrial chain of the manufacturing industry. Manufacturing sectors and productive service sectors complement each other well, which helps to increase production efficiency in manufacturing sectors and encourages its growth to the top of production value chain [2, 3]. Despite being a major industrial nation, China still lags far behind wealthy nations in global value chain, mostly due to low technological advancement and a lack of R&D funding [4]. Therefore, in order to improve the core competitiveness of manufacturing industries, China needs to enhance the interaction between industries. The collaborative agglomeration of manufacturing and productive service industries is conducive to reducing transaction costs and promoting the transformation of technological innovation. It improves the value chain correlation between upstream industries and downstream industries, and exchanges knowledge, information, and technology between industries [5]. Industrial collaborative agglomeration is crucial for China to overcome the current manufacturing development dilemma. Additionally, it is a crucial means to achieve high-quality economic growth, to promote economic structural upgrade, and to optimize industrial layout [6].

The 1990s witnessed the emergence of digital economy, which is a new economy based on human intelligence networking, and the digital revolution in information technology [7]. Digital economy has grown rapidly due to the digital transformation of various industrial variables and the advancement of information and communication technology [8]. Modern information network has become a main carrier for changing the global competitive landscape. Currently, China’s digital economy is experiencing tremendous expansion. As reported by China Digital Economy Development Report (2023), in 2022, China’s digital economy reached 50.2 trillion yuan, a nominal increase of 10.3% year-on-year, accounting for 41.5% of GDP. Among it, 9.2 trillion yuan was from digital industrialization and 41 trillion yuan from industrial digitalization, representing 18.3% and 81.7% of the total digital economy, respectively. It is evident that digital economy plays a crucial role in China’s economic development. In recent years, governments worldwide have implemented plans to promote the growth of digital economy. The integration of digital technology across various industries can provide a new stimulus for their economic development and integration. At the same time, China’s ‘14th Five-Year Plan’ emphasizes the role of digital economy as a product of the new scientific and technological revolution. Digital economy accelerates the fission and aggregation of industrial factors, leading to a more accurate and agglomerated allocation of production and service resources. Digital economy helps to transform and upgrade traditional manufacturing industries to become more intelligent. Additionally, it can encourage the growth of profitable service and manufacturing industries. In recent years, it is believed that digital economy is innovating the form of industrial organization, blurring industrial boundaries, and reshaping the mode of industrial collaborative interaction [9].

Ellison first proposed the concept of industrial collaborative agglomeration, which suggests that labor sharing, factor costs, and technology spillovers are crucial in promoting the formation of geospatial collaborative agglomeration across different industries [10]. Since then, numerous scholars have extensively discussed the topic of industrial collaborative agglomeration. On the one hand, researchers have measured the level of industrial collaborative agglomeration using various models, including the industry-demographic matching degree and the coupled coordination model. These models aim to assess the degree of collaborative agglomeration between different industries [11–13]. On the other hand, regarding the impact of industrial collaborative agglomeration, previous researches have demonstrated that industrial collaborative agglomeration is a crucial method to advance the industrial chain to the middle and high end [14], to enhance enterprise production efficiency, to promote industrial structure upgrading [15, 16], to improve regional total factor productivity [17] and to raise urbanization level [18]. Ultimately, this leads to the high-quality development of economy [19, 20]. For instance, industrial collaborative agglomeration leads to spatial agglomeration, generating knowledge spillover effects that adjust industrial structure and enhance regional innovation. This, in turn, improves the local economy’s risk-resistant ability and promotes high-quality economic development [21]. Simultaneously, with the rapid growth of digital economy, academics have shifted their research focus towards the impact of the digital economy on industrial development. For example, Chen and Yang analyze the impact of the digital economy on the upgrading of industrial structure from two perspectives: digital industrialization and industrial digitization. They conclude that the digital economy significantly facilitates industrial structure upgrading [22]. With an emphasis on sustainable urban development, Yin et al. show how digital innovation in construction management may promote industrial upgrading and argue that the expansion of digital economics facilitates the creation of sustainable urban solutions [23]. Yin et al. also demonstrate how digital innovation can result in the sustainable transformation of manufacturing processes, highlighting green and intelligent manufacturing practices to broaden digital economy [24]. In addition, the existing literature reveals that digital economy has a significant positive impact on the transformation and upgrading of industrial structure from a variety of perspectives, including enterprise digital transformation, capital and labour allocation efficiency, technology spillover effect, innovation and entrepreneurship, and human capital [25–29]. At the same time, some studies have also analyzed the impact of digital economy on economic development from a variety of perspectives, including green technology innovation, industrial structure, employment structure, and human capital [30–35]. Han and Li argue that the development of high-speed railways promotes industrial collaborative agglomeration in the region [36]. Similarly, Guo and Yuan suggest that the Internet, as a new-generation information technology, significantly reshapes the collaborative agglomeration of urban industries [37].

There are abundant studies on the impact of digital economy. However, there are few studies in the literature that have analyzed the impact of digital economy on industrial collaborative agglomeration. Only a few literature has examined the positive impact of the digital economy on industrial collaborative agglomeration from the perspective of high-speed railway and Internet development. Based on previous studies, it can be found that the effect mechanism of digital economy on industrial collaborative agglomeration in China and the economic relations between them are still not investigated. Moreover, there is a clear lack of research on the role and of government intervention and entrepreneurial activity in promoting the relationship between the development of digital economy and industrial collaborative agglomeration. This study integrates digital economy and industrial collaborative agglomeration between manufacturing industries and productive service industries and fills the research gap in digital economy and industrial development area. It innovatively analyzes the non-linear impact of digital economy on industrial collaborative agglomeration, and tries to find whether the impact of digital economy is different in different areas of China. Moreover, based on moderating effect and mediating effect model, it creatively explores how government intervention and entrepreneurial activity affects industrial collaborative agglomeration through digital economy. In addition, whether the impact of digital economy on industrial collaborative agglomeration has heterogeneous effects in different industries is also investigated. A panel data set from 286 prefecture-level cities in China between 2011 and 2021 is employed and the Tobit model is applied to conduct the analysis. This study contributes to from three aspects. Firstly, the non-linear relationship between digital economy and industrial collaborative agglomeration can reveal the different impacts of digital economy at different stage. It provides references for policy makers to further understand the impact path of digital economy and to make relevant specific policies to promote digital economy and industrial collaborative agglomeration at different stages. Secondly, the finding of heterogeneous effects in different regions of China and in different levels of industries can provide guidance for the formulation of regional development policies and industrial development policies of digital economy according to local conditions. Thirdly, to explain the interaction role of government intervention and entrepreneurial activity in digital economy to promote industrial collaborative agglomeration can give implications for government and enterprises to understand their roles more clearly and provide an important theoretical basis for building a digital economy innovation community in China.

## 2. Research methodology and data

### 2.1 The impact of digital economy on industrial collaborative agglomeration

**Fig 1:**
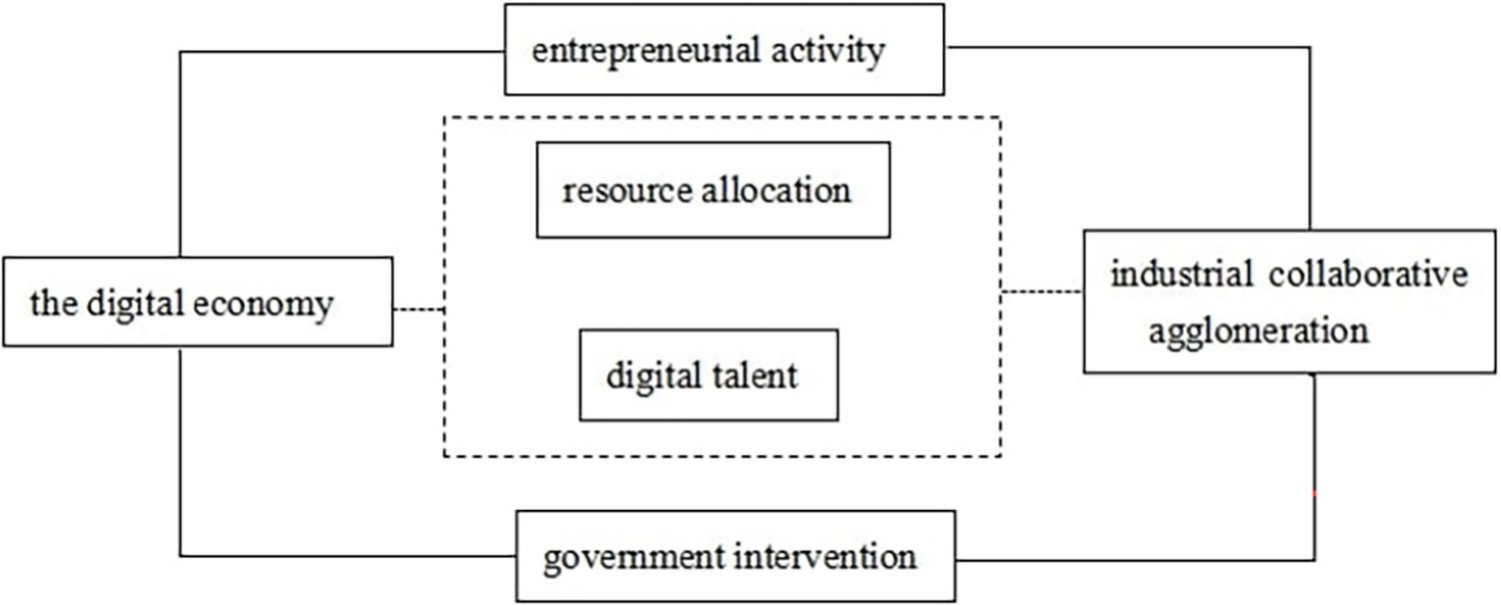
The impact path of the digital economy on industrial collaborative agglomeration.

Digital economy, a new economic form that utilizes data resources as elements and modern networks as carriers, can lower industrial production costs and facilitate industrial collaborative agglomeration [38, 39]. However, achieving industrial collaborative agglomeration through digital economy is not a simple task, as it can be affected by resource mismatch and data security. Digital economy also has a significant impact on labour market by optimizing industrial structure. The integration of digital economy into industrialization process requires a large number of professionals with proficiency in digital technology, industry experience, and digital skills. This may result in the elimination of professionals with non-digital skills, such as packaging and handling [40, 41]. In early stages, due to a lack of digital talents, local industries may struggle to effectively apply digital technology to their production processes. This can disrupt the distribution of talents between industries and hinder industrial collaborative agglomeration. In middle and late stages, a large number of digital technology experts continue to join enterprises, creating favourable conditions for collaborative industrial agglomeration [42]. Furthermore, the development of digital economy are not be evenly distributed across all industries. Generally speaking, digital technology are firstly applied in manufacturing industries due to their higher output value. Then, through technology spillover, digital economy promotes the development of other industries [43]. Higher output value of manufacturing industries attracts more high-quality resources, leading to the prosperity of manufacturing industries yet the shrinkage of productive service industries. The increasing integration of digital technology across all industries is expected to reshape industrial structure, and to promote industrial collaborative agglomeration between manufacturing industries and productive service industries. Fig 1 shows the impact path of digital economy on industrial collaborative agglomeration. Based on the above analysis, this research proposes the following hypothesis H1.

**H1:** Digital economy has a significant U-shaped relationship with collaborative agglomeration of manufacturing and productive services industries in China.

### 2.2 The moderating effect of government intervention

The relationship between government and market is one of the core issues in China. To balance the relationship between government and market is conducive to upgrade economic structure and promote high-quality economic development in China [44, 45]. During the development of digital economy, problems such as insufficient information, behaviour bias, market blindness, and weak profitability can lead to market failure and inefficient allocation of resources [46, 47]. However, government can effectively alleviate market failures and create a favorable environment for the collaborative agglomeration of regional industries by investing in digital infrastructure construction and maintaining market competition order [48]. Based on the above analysis, this research proposes the following hypothesis H2.

**H2:** Government intervention has a moderating effect on the influence of digital economy on industrial collaborative agglomeration.

### 2.3 The mediating effect of entrepreneurial activity

Digital economy provides increased entrepreneurial opportunities by optimizing the combination of production factors, and by expanding market size and knowledge spillover effects. It can also enrich entrepreneurial resources by accelerating information dissemination, thereby promoting urban entrepreneurial activity [49]. Moreover, the growth of digital economy alters product delivery, increases various consumer demands, expands product number and variety, and serves as a foundation for entrepreneurial endeavors [50]. In addition, digital economy is gradually making information exchange platforms more convenient. This can provide entrepreneurs with a source of information and a base for their entrepreneurial activities. Besides, product diversification and scale expansion can lead to information redundancy, yet digital economy can optimize information matching process, reduce information redundancy, and provide a solid foundation for entrepreneurship [51].

The increase of entrepreneurial activity also contributes to optimizing industrial structure and promoting industrial collaborative agglomeration [52, 53]. Innovation and entrepreneurship are crucial catalysts for the vitality of market economy. The positive and creative initiative of entrepreneurs can integrate innovative resources, optimize resource allocation, and create favourable conditions for industrial collaborative agglomeration [54]. The entrepreneurial activities of successful entrepreneurs can attract a large number of entrepreneurs to learn and imitate, thereby increasing entrepreneurial activity and promoting industrial collaborative agglomeration [55]. What is more, entrepreneurs can overcome resource constraints through social network relationships. This allows them to obtain more resources, information, and support. At the same time, social networks provide critical complements to entrepreneurial and innovative decision-making, and help bind different actors within the industrial agglomeration [56]. Based on the above analysis, this research proposes the following hypothesis H3.

**H3:** The digital economy significantly encourages industrial collaborative agglomeration by fostering entrepreneurial activity.

### 2.4 Model

#### (1) Non-linear regression model

As the index of collaborative industrial agglomeration is non-negative truncated data, ordinary OLS regression may not apply to analyze the data. Based on previous studies, Tobin [57] believes that Tobit model can effectively deal with non-negative truncated data. Therefore, this study adopts the Tobit estimation method of Wu et al [58] and selects digital economy quadratic term to construct the Tobit model, and the specific model is set as follows:

$$\begin{array}{c} COAGGLO_{it}=\alpha_{0}+\alpha_{1}DIGE_{it}+\alpha_{2}DIGE_{it}^{2}+\alpha_{3}\ln HUM_{it}+\alpha_{4}IS_{it}\\ +\alpha_{5}\ln GDP_{it}+\alpha_{6}URB_{it}+\alpha_{7}LM_{it}+\varepsilon_{it} \end{array}$$ (Eq 1)

Here COAGGLO represents the industrial collaborative agglomeration index of each city. DIGE refers to digital economy. DIGE^2^ is the quadratic term of digital economy and is used to examine the non-linear relationship. HUM denotes human capital. IS stands for industrial structure, GDP for economic development, URB for urbanization rate, and LM for labour mobility. This model tests the hypothesis H1.

#### (2) Moderating effects model

The concept of moderating effect was first proposed by Aiken and West [59]. Since then, it has become one of the most commonly used analysis methods in social science research. This model here tests hypothesis H2 by using the research method of Wu et al.[60], which introduces the quadratic term of digital economy (DIGE^2^) and its interaction term, government intervention (GM). The model to test hypothesis H2 is set as follows:

$$\begin{array}{c} COAGGLO_{it}=\beta_{0}+\beta_{1}DIGE_{it}+\beta_{2}DIGE_{it}^{2}+\beta_{3}DIGE_{it}\cdot GM_{it}+\beta_{4}DIG{E^{2}}_{it}\cdot GM_{it}+\beta_{5}GM_{it}\\ +\beta_{6}\ln HUM_{it}+\beta_{7}IS_{it}+\beta_{8}\ln GDP_{it}+\beta_{9}URB_{it}+\beta_{10}LM_{it}+\varepsilon_{it} \end{array}$$ (Eq 2)

In Eq 2, GM_it_ refers to government intervention. DIGE·GM indicates the moderating effect of government intervention with digital economy. DIGE^2^·GM stands for the moderating effect of government intervention with the quadratic term of digital economy.

#### (3) Mediation effects model

To test hypothesis H3, this study employs Li and Liu’s research method to construct a mediation effect model [61]. The model employs entrepreneurial activity (EA) as a mediator variable to explore the impact path of digital economy on industrial collaborative agglomeration. The specific model is set as follows:

$$\begin{array}{c} COAGGLO_{it}=\alpha_{0}+\alpha_{1}DIGE_{it}+\alpha_{2}DIGE_{it}^{2}+\alpha_{3}\ln HUM_{it}+\alpha_{4}IS_{it}\\ +\alpha_{5}\ln GDP_{it}+\alpha_{6}URB_{it}+\alpha_{7}LM_{it}+\varepsilon_{it} \end{array}$$ (Eq 3)

$$\begin{array}{c} EA_{it}=\gamma_{0}+\gamma_{1}DIGE_{it}+\gamma_{2}DIGE_{it}^{2}++\gamma_{3}\ln HUM_{it}+\gamma_{4}IS_{it}\\ +\gamma_{5}\ln GDP_{it}+\gamma_{6}URB_{it}+\gamma_{7}LM_{it}+\varepsilon_{it} \end{array}$$ (Eq 4)

$$\begin{array}{c} COAGGLO_{it}=\pi_{0}+\pi_{1}DIGE_{it}+\pi_{2}DIGE_{it}^{2}+\pi_{3}EA_{it}+\pi_{4}\ln HUM_{it}+\pi_{5}IS_{it}\\ +\pi_{6}\ln GDP_{it}+\pi_{7}URB_{it}+\pi_{8}LM_{it}+\varepsilon_{it} \end{array}$$ (Eq 5)

In Eqs 3, 4 and 5, EA_it_ represents entrepreneurial activity. The other variables are in line with Eqs 1 and 2.

### 2.5 Variable explanation and data sources

#### (1) Industrial collaborative agglomeration

This research focuses on industrial collaborative agglomeration (COAGGLO) as the explained variable. The measurement of COAGGLO is based on the study of Zheng and He [2]. The industrial collaborative agglomeration index reflects the degree of agglomeration between manufacturing industries and productive service industries, such as transportation, warehousing, and postal industry. The index is calculated based data from each city’s statistical yearbook. The calculation equation is as follows:

$$MOAGGLO_{it}=(E_{im}/\sum_{i}E_{im})/(\sum_{m}E_{im}/\sum_{i}\sum_{m}E_{im})$$ (Eq 6)

$$POAGGLO_{it}=(E_{ip}/\sum_{i}E_{ip})/(\sum_{p}E_{ip}/\sum_{i}\sum_{p}E_{ip})$$ (Eq 7)

In Eqs 6 and 7, E_im_ and E_ip_ represent the number of employees in manufacturing and productive service industries in city i, respectively. MOAGGLO_it_ and POAGGLO_it_ indicate the calculated index of regional manufacturing and productive service industries by the Entropy method in each city of China. The productive service sectors include transport, storage, postal, financial, scientific, technical, and geological survey services, wholesale and retail commerce, information transmission, computer services and software, leasing, and business services.

$$COAGGLO_{it}=(\alpha (1-\frac{\left|MOAGGLO_{it}-POAGGLO_{it}\right|}{MOAGGLO_{it}+POAGGLO_{it}})+\alpha (\theta_{1}MOAGGLO_{it}+\theta_{2}POAGGLO_{it}))$$ (Eq 8)

Where COAGGLO_it_ represents the industrial collaborative agglomeration index of the city i at the time t. $\left(1-\frac{\left|MOAGGLO_{it}-POAGGLO_{it}\right|}{MOAGGLO_{it}+POAGGLO_{it}}\right)$ is the collaborative quality between the two types of industries, and $\left(\theta_{1}MOAGGLO_{it}+\theta_{2}POAGGLO_{it}\right)$ is the collaborative height between the two types of industries. To reflect the heterogeneity of industry segmentation and correct the collaborative ’inflated’ phenomenon, this research uses weighting coefficients for the proportion of the value-added of the secondary industry in the GDP of each city (*θ*_*1*_) and the proportion of the value-added of the tertiary industry in the GDP of each city (*θ*_*2*_).

#### (2) Digital economy

The core explanatory variable is digital economy (DIGE). Li et al. measured the level of digital economy from three different aspects: digital informatization, Internet development, and digital transaction [62]. Zhang et al. measured the level of digital economy in terms of digital industry growth, innovation and digitization capacity, and digital technology application [63]. Liu et al. constructed a digital economy index by using five indicators: Internet penetration, Internet employees, Internet output, Internet penetration rate, and digital financial inclusion [64]. As the sample for this study is urban data, this research uses the research method of Liu et al. [64] to construct a digital economy index system. The Entropy method is applied to calculate the level of urban digital economy. The specific indicators of the digital economy used in this research are shown in Table 1.

**Table 1:** Indicators for evaluating the level of digital economy.

	Indicators	Meaning	Source
Digital Economy Development Index	Internet penetration	Internet broadband access per 100 population	Statistical yearbook
	Internet-related practitioners	Number of employees in the computer services and software industry as a share of employees in urban units	Statistical yearbook
	Internet-related outputs	Total telecommunication services per capita	Statistical yearbook
	Cell phone penetration rate	Cell phone subscriptions per capita	Statistical yearbook
	Digital Inclusive Finance Index	Peking University Digital Inclusive Finance Index	Peking University Financial Database

The specific calculation steps based on the entropy method are as follows:

Data standardization:

$$\begin{array}{l} {M^{\prime }}_{i,j}=\frac{M_{i,j}-\min(M_{i,j})}{\max(M_{i,j})-\min(M_{i,j})}(\mathrm{Positive}\;\mathrm{indicators})\\ {M^{\prime }}_{i,j}=\frac{\min(M_{i,j})-M_{i,j}}{\max(M_{i,j})-\min(M_{i,j})}(\mathrm{Negative}\;\mathrm{indicators})\\ i=1,2\cdots m;j=1,2\cdots n \end{array}$$ (Eq 9)

In Eq (9), M_ij_ is the result of standardizing the data and represents the j^th^ indicator in year i.

Calculate the weight of the j^th^ indicator in year i:

$$P_{i,j}={M^{\prime }}_{i,j}/\sum_{i=1}^{x}{M^{\prime }}_{i,j}$$ (Eq 10)

Calculate the information entropy of the j^th^ metric:

$$R_{j}=-1/\ln(x)\sum_{i=1}^{x}\left\{P_{i,j}\ln(P_{i,j})\right\}$$ (Eq 11)

Calculate the information entropy redundancy:

$$D_{j}=1-R_{j}$$ (Eq 12)

Calculation of indicator weights:

$$W_{i}=D_{j}/\sum_{i=1}^{n}D_{j}$$ (Eq 13)

Calculation of the finanal index:

$$V=\sum_{j=1}^{n}W_{i}{M^{\prime }}_{ij}$$ (Eq 14)

#### (3) Entrepreneurial activity

The mediating variable in this research is entrepreneurial activity (EA). Combined with the availability of data at the city level in China, the entrepreneurial activity is indicated by the ratio of the number of urban private enterprise employees and the number of self-employed business employees to the total number of employees in each prefecture-level city [65]. The data source of entrepreneurial activity is each city’s statistical yearbook of China.

#### (4) Government intervention

The moderating variable in this study is government intervention (GM), which is measured as the government expenditure budget as a proportion of GDP [66]. The data source of this variable is the Mark’s Database (www.macrodatas.cn)

#### (5) Other control variables

To eliminate the influence of other factors on industrial collaborative agglomeration, this study selects five control variables based on Dong et al. and Wang and Li [67, 68]: namely, human capital (HUM), industrial structure (IS), economic development (GDP), urbanization rate (URB), and labor mobility (LM). Human capital (HUM) shows the education level of each city, is indicated by the number of students enrolled in general institutions of higher education in each city. Industrial structure (IS) is expressed as the ratio of the value added of the secondary industry to the value added of the tertiary industry in each city. Economic development level (GDP) is represented by GDP per capita of each city. Urbanization rate (URB) is denoted by the ratio of urban population to the city’s total population. Labor mobility (LM) is shown as the ratio of primary industry employment to total employment in each city. The data source of human capital, GDP per capita and urbanization rate is each city’s statistical yearbook. The data source of industrial structure and labor mobility is the Mark’s Database (www.macrodatas.cn).

## 3. Empirical results

### 3.1 Descriptive statistics and correlation test

The descriptive statistics of each variable is reported in Table 2. For the missing data, interpolation method is used to make up. Table 3 shows that all correlation regression coefficients are less than 0.7, which suggests that the model does not contain multicollinearity [69]. The variance inflation factor is 1.75, which is less than 10, which further confirms that there is no multicollinearity in the research model.

**Table 2:** Results of descriptive statistics.

Variable	sample size	mean	standard deviation	minimum value	maximum values
COAGGLO	3135	0.048	0.020	0.011	0.461
DIGE	3135	0.026	0.035	0.002	0.667
GM	3135	0.205	0.123	0.044	2.364
EA	3135	1.332	1.258	0.023	48.046
lnHUM	3135	10.566	1.371	4.511	13.957
IS	3135	1.187	0.598	0.190	8.848
lnGDP	3135	10.754	0.571	8.773	13.055
URB	3135	0.044	0.035	0.000	0.315
LM	3135	0.025	0.144	0.000	7.321

**Table 3:** Correlation test results.

	COAGGLO	DIGE	DIGE2	lnHUM	IS	lnGDP	URB	LM	EA	GM
COAGGLO	1									
DIGE	0.349***	1								
DIGE2	0.290***	0.685***	1							
lnHUM	0.382***	0.213***	0.073***	1						
IS	-0.060***	-0.205***	-0.057***	-0.254***	1					
lnGDP	0.475***	0.354***	0.154***	0.396***	-0.026	1				
URB	0.369***	0.232***	0.150***	0.347***	-0.047***	0.275***	1			
LM	0.232***	-0.013	-0.012	-0.067***	-0.045**	-0.066***	-0.131***	1		
EA	0.316***	0.119***	0.063***	-0.039**	-0.146***	0.094***	0.018	0.574***	1	
GM	0.325***	0.321***	0.143***	0.633***	-0.404***	0.462***	0.448***	-0.104***	0.075***	1

Note

*, **, and *** indicate p<0.1, p<0.05, and p<0.01, respectively

### 3.2 Regression results

The effects of digital economy on urban industrial collaborative agglomeration are presented in Table 4. Without the addition of control variables, column (1) demonstrates that the influence of the digital economy (DIGE) on industrial collaborative agglomeration is negative and significant, whereas the coefficient of DIGE^2^ is positive and significant. This suggests that the relationship between digital economy and urban industrial collaborative agglomeration is U shaped. In order to further validate the relationship between digital economy and industrial collaborative agglomeration, results in column (2) to (6) are reported to show the regression coefficients of each control variable. At a 1% significance level, the coefficients of DIGE^2^ are considerably positive, while the coefficients of DIGE are significantly negative. This verifies that the impact of digital economy on urban industrial collaborative agglomeration has a U-shaped relationship. At the same time, all models are further tested by U-test method to ensure that the data do not store a kind of monotonous curve, which proves that there is indeed a strict U-shaped relationship between digital economy and industrial collaborative agglomeration.

**Table 4:** Tobit regression results of the whole sample.

Variables	(1)	(1)	(2)	(3)	(4)	(5)
	COAGGLO	COAGGLO	COAGGLO	COAGGLO	COAGGLO	COAGGLO
DIGE	-0.113***	-0.130***	-0.119***	-0.146***	-0.148***	-0.158***
	(-7.228)	(-8.207)	(-7.018)	(-8.095)	(-8.231)	(-11.15)
DIGE^2^	0.435***	0.465***	0.445***	0.490***	0.486***	0.511***
	(13.60)	(14.34)	(13.10)	(13.80)	(13.67)	(18.35)
lnHUM		3.32e-08***	3.42e-08***	3.09e-08***	2.67e-08***	2.46e-08***
		(7.131)	(7.342)	(6.882)	(6.042)	(5.464)
IS			0.00110**	0.00172***	0.00161***	0.00121***
			(1.962)	(2.981)	(2.818)	(2.609)
lnGDP				0.00441***	0.00452***	0.00306***
				(4.504)	(4.677)	(3.774)
URB					0.0920***	0.0723***
					(4.239)	(3.089)
LM						0.0578***
						(42.19)
Constant	0.0506***	0.0477***	0.0461***	-0.00117	-0.00571	0.0103
	(48.23)	(46.76)	(34.67)	(-0.111)	(-0.550)	(1.164)
Observations	3,135	3,135	3,135	3,135	3,135	3,135

Note

*, **, and *** indicate p<0.1, p<0.05, and p<0.01, respectively; numbers in parentheses are t-values.

**Fig 2:**
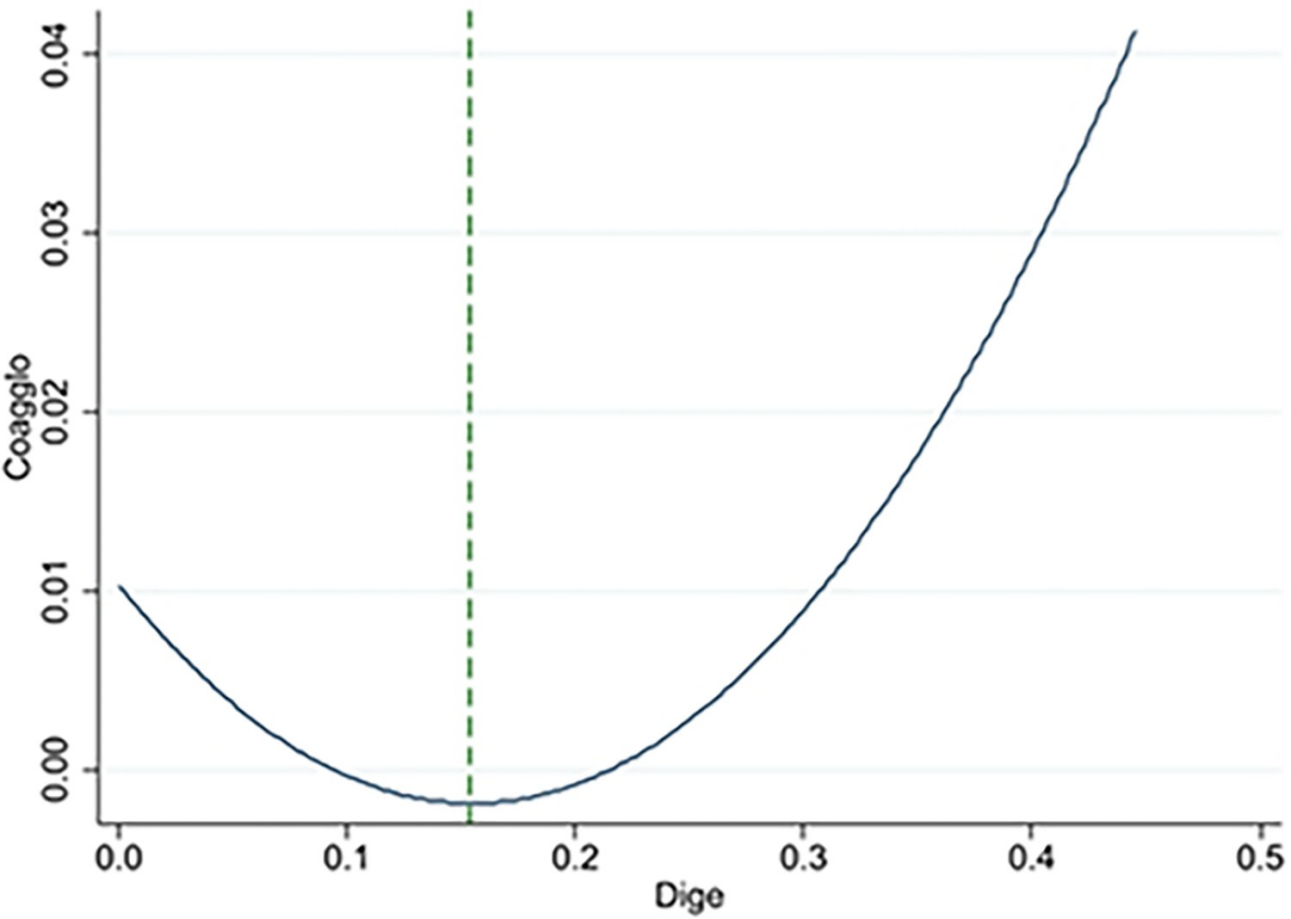
Relationship between digital economy and industrial collaborative agglomeration.

Fig 2 illustrates the U-shape test results. The extreme point is 0.154, within the value range of [0.002, 0.667] of the digital economy. It shows that digital economy has a negative slope to the left of the extreme point and a positive slope to the right of the extreme point. It further proves that the U-shaped relationship between digital economy and urban industrial collaborative agglomeration is significant, thus verifying the hypothesis H1. Moreover, the coefficient of human capital (lnHUM) is significantly positive, which indicates that the improvement of human capital increases the supply of digital technology talents, and then promotes industrial collaborative agglomeration. The industrial structure (IS) coefficient is highly positive, suggesting that industrial collaborative agglomeration can be successfully driven by industrial structure upgrading. The significantly positive coefficient of economic development level (lnGDP) implies that economic growth facilitates industrial collaborative agglomeration. The regression coefficient of urbanization rate (URB) is significantly positive, which means that urbanization is conducive to the promotion of industrial collaborative agglomeration. The coefficient of labor mobility (LM) is significantly positive. It implies that the concentration of talents is an important factor for industrial collaborative agglomeration, and the mobility of labor forces promotes industrial collaborative agglomeration.

### 3.3 Heterogeneity results

#### (1) Industry heterogeneity

**Fig 3:**
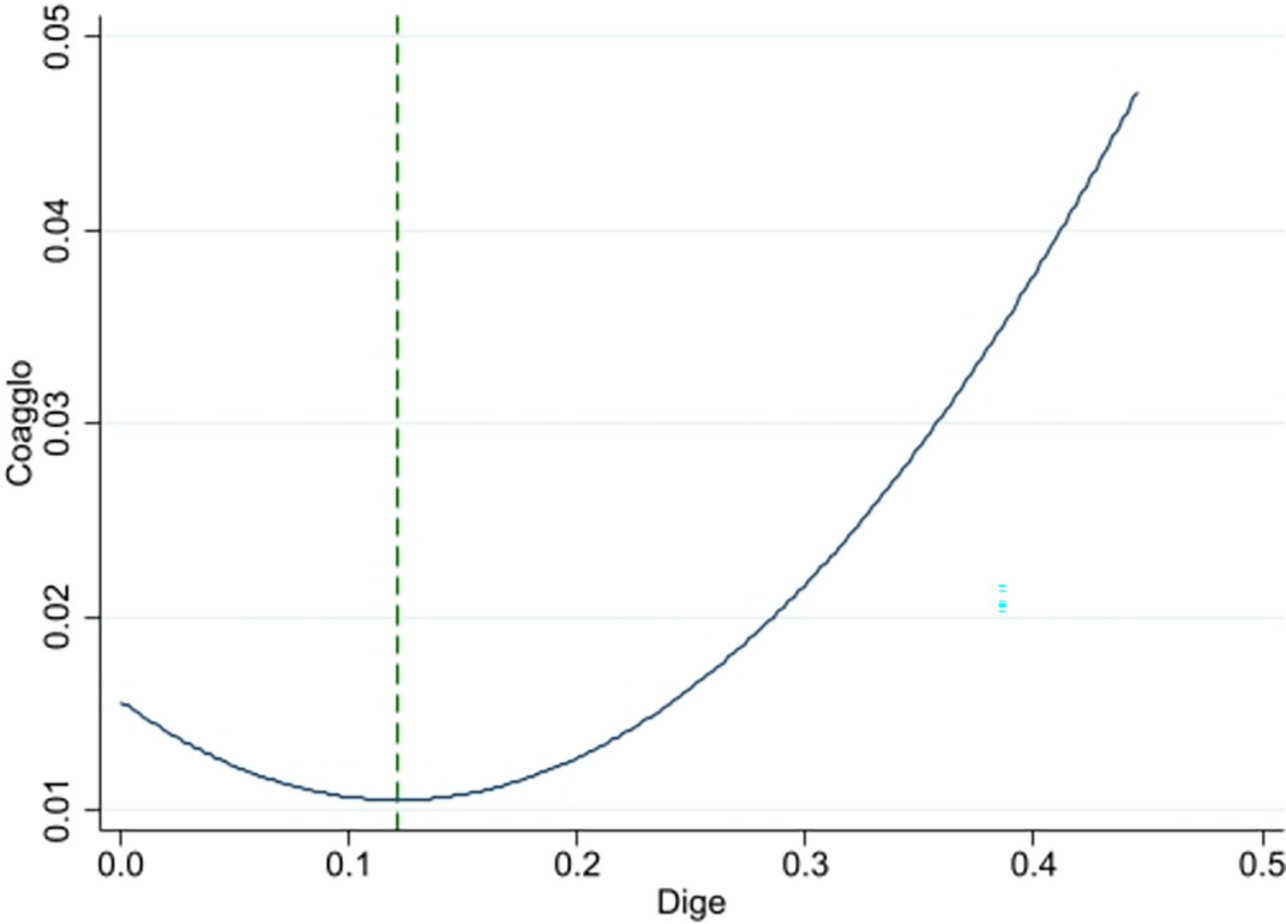
Relationship between digital economy and collaborative agglomeration in high-end industries.

**Fig 4:**
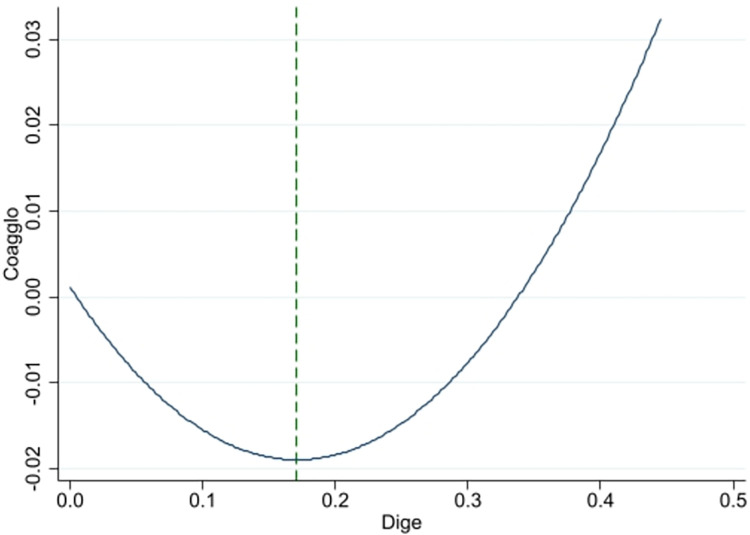
Relationship between digital economy and collaborative agglomeration in middle- and low-end industries.

According to the White Paper on China’s Digital Economy Development and Employment (2022), information transmission, software and information technology service industry, financial industry, and scientific research and technology service industry in productive service sectors have a higher proportion in digital economy. These industries are an important force in promoting the digital transformation of China’s industries. Thus this research categorizes information transmission, software and information technology services, finance, and scientific research and technology services as high-end productive services in digital economy, and wholesale and retail business, transportation, warehousing and postal services, and leasing and business services as middle- and low-end productive services. Table 5 displays the regression results in column (1) and (2). Figs 3 and 4 show the relationship between the digital economy and collaborative agglomeration in high-end industries, and in middle- and low-end industries respectively. The significant negative coefficient of DIGE and positive coefficient of DIGE^2^ in column (1) and column (2) suggest a U-shaped relation between digital economy and industrial collaborative agglomeration both in high-end, and middle-and low-end industries. The results also indicate that digital economy promotes collaborative agglomeration not only in high-end productive services and manufacturing industries, but also in middle- and low-end productive services and manufacturing industries. The regression U-shaped inflection point of high-end productive service and manufacturing industries is 0.120, while the inflection point of middle- and low-end productive service and manufacturing industries is 0.171. This indicates that digital economy promotes collaborative agglomeration in high-end productive service and manufacturing industries earlier than in middle- and low-end productive service and manufacturing industries.

**Table 5:** Industry heterogeneity of digital economy on industrial collaborative agglomeration.

Variables	(1)	(2)
	COAGGLO of high-end productive service and manufacturing industries	COAGGLO of low- and middle-end productive services and manufacturing industries
DIGE	-0.0844***	-0.234***
	(-6.914)	(-12.37)
DIGE^2^	0.349***	0.683***
	(14.53)	(18.36)
lnHUM	1.68e-08***	3.60e-08***
	(4.450)	(6.170)
IS	0.00244***	-0.000185
	(6.096)	(-0.299)
lnGDP	0.00186***	0.00456***
	(2.675)	(4.285)
URB	0.0661***	0.0870***
	(3.450)	(2.876)
LM	0.0540***	0.0619***
	(45.65)	(33.84)
Constant	0.0156**	0.00106
	(2.058)	(0.0918)
Observations	3,135	3,135

Note

*, **, and *** indicate p<0.1, p<0.05, and p<0.01, respectively; numbers in parentheses are t-values.

#### (2) Regional heterogeneity

Due to the obvious differences in the development stage and resource allocation of each region in China, the development of digital economy and industrial collaborative agglomeration are heterogeneous in different regions. At the same time, the impact of the digital economy on industrial collaborative agglomeration in different regions also has heterogeneity. To examine the specific regional heterogeneity, this research divides the whole sample into three major regions of eastern, central and western of China, and applies data regression respectively. The results are shown in Table 6. Based the regression results in Table 6, the impact of digital economy on industrial collaborative agglomeration has obvious regional heterogeneity. The estimated coefficients of DIGE (digital economy) in the central region and western region are significantly negative, and the estimated coefficients of DIGE^2^ are significantly positive. However, the coefficients of DIGE and DIGE^2^ in the eastern region are not significant. This indicates that the digital economy in the central and western regions plays a better role in promoting the collaborative agglomeration of industries.

**Table 6:** Regional heterogeneity regression results.

Variables	(1) eastern region	(2) central region	(3) western region
	COAGGLO	COAGGLO	COAGGLO
DIGE	-0.0218	-0.183***	-0.366***
	(-1.323)	(-2.985)	(-13.30)
DIGE2	0.0387	0.944*	1.242***
	(1.183)	(1.700)	(25.60)
lnHUM	2.66e-08***	1.50e-08**	3.83e-08***
	(3.222)	(2.564)	(4.874)
IS	0.00524***	-0.000618	0.000883
	(4.685)	(-1.055)	(1.314)
lnGDP	-0.00421***	0.00167	0.00385***
	(-3.143)	(1.561)	(2.785)
URB	0.0819***	0.143***	-0.0508
	(2.842)	(3.525)	(-0.728)
LM	0.00766	0.0597***	-0.0252
	(0.285)	(61.72)	(-1.362)
Constant	0.0895***	0.0216**	0.00526
	(5.954)	(1.962)	(0.351)
Observations	1,100	1,199	836

Note

*, **, and *** indicate p<0.1, p<0.05, and p<0.01, respectively; numbers in parentheses are t-values.

At the same time, the regression U-inflection point in the central region is 0.096, while the regression U-inflection point in the western region is 0.147, indicating that digital economy in the central region reaches the right side of the U-inflection point earlier, and promotes the collaborative industrial agglomeration earlier. This is because the eastern region has better economic development, more advanced productive services and manufacturing industries, and higher industrial collaborative agglomeration level. According to the law of diminishing marginal effect, the impact of digital economy on industrial collaborative agglomeration in the eastern region are not obvious. The productive services and manufacturing industries in the western region are mainly labor-intensive. There is still a large improvement space for the western region to develop the collaborative agglomeration between productive services industries and manufacturing industries.

### 3.4 Robustness and endogeneity tests

#### 3.4.1 Robustness tests

First, to test the robustness of the empirical results above, this research employs the principal component analysis to calculate the digital economy index again, and uses the new calculated data to conduct the data analysis once more (see Table 7, column (1)). It directly uses the digital inclusive finance index to represent the digital economy index to examine the impact of digital economy on industrial collaborative agglomeration (see Table 7, column (2)). Both regression results in column (1) and (2) indicate similar results: that digital economy has a negative impact on industrial collaborative agglomeration in the beginning and then a positive impact, indicating a U shaped relationship. Second, this research applies a two-way fixed-effects estimation method to conduct the data analysis again, and the results are shown in Table 7, column (3). It also shows similar results that the relationship between digital economy and industrial collaborative agglomeration is a U shape. Moreover, to avoid interference with the regression results due to outliers in the sample, this research uses the method of Bettis et al. [65] to shrink all variables and conduct the regression once more. The results are shown in column (4) of Table 7, which also implies a U shaped relation between digital economy and industrial collaborative agglomeration. All the regression results in Table 7 from column (1) to column (4) indicate the robustness of the empirical results in Section 3.2.

**Table 7:** Robustness and endogeneity test results.

Variables	(1)	(2)	(3)	(4)	(5)
	COAGGLO	COAGGLO	COAGGLO	COAGGLO	COAGGLO
DIGE	-0.113*	-0.000142***	-0.160***	-0.0864***	
	(-1.721)	(-11.08)	(-9.690)	(-3.052)	
L. DIGE					-0.263***
					(-9.836)
DIGE ^2^	0.118***	2.39e-07***	0.529***	0.574***	
	(2.645)	(7.064)	(17.65)	(3.018)	
L. DIGE^2^					1.515***
					(18.17)
lnHUM	2.08e-08***	1.73e-08***	-5.66e-09	1.46e-08***	2.73e-08***
	(4.659)	(4.184)	(-0.791)	(3.531)	(6.422)
IS	0.00204***	-0.00248***	-0.000485	0.00171***	0.000180
	(4.327)	(-4.003)	(-0.784)	(3.908)	(0.339)
lnGDP	9.19e-09	0.0105***	0.00268**	-0.000498	0.00637***
	(0.720)	(9.562)	(2.300)	(-0.714)	(7.198)
URB	0.159***	0.145***	-0.234***	0.174***	0.0830***
	(7.395)	(7.190)	(-5.863)	(7.242)	(3.836)
LM	0.0570***	0.0564***	0.0581***	-0.000636	0.0576***
	(38.62)	(38.61)	(44.17)	(-0.0982)	(42.64)
Constant	0.0588**	-0.0539***	0.00607	0.0441***	-0.0244**
	(2.481)	(-4.902)	(0.518)	(5.955)	(-2.566)
Observations	3,135	3,135	3,135	3,135	2,850

Note

*, **, and *** indicate p<0.1, p<0.05, and p<0.01, respectively; numbers in parentheses are t-values.

#### 3.4.2 Endogeneity tests

There might be an endogeneity problem in the research model, such as a reverse causality relation between digital economy and industrial collaborative agglomeration. In order to avoid this endogeneity problem of reverse causality, this research employs a lag period of DIGE and DIGE^2^ to conduct the regression analysis again. The results are reported in column (5) of Table 7. It shows that the lag term of DIGE also has a significant negative impact and the lag term of DIGE^2^ has a significant positive impact on industrial collaborative agglomeration, This implies a U shaped relationship between digital economy and industrial collaborative agglomeration and no endogenous problem among the variables.

### 3.5 Test of moderating effect and mediation effect

Based on previous theoretical analysis, it is concluded that government intervention is conducive to regulating the role of digital economy in industrial collaborative agglomeration. Therefore, this study introduces the interaction term of government intervention and digital economy to further reveal the impact mechanism of digital economy on industrial collaborative agglomeration.

The moderating regression results are shown in column (1) of Table 8. The interaction term between government intervention and digital economy (GM×DIGE) is positive at 1% significance. It indicates that active government inhibits the negative impact of digital economy on industrial collaborative agglomeration and has an important role in the era of digital economy. The interaction term GM×DIGE^2^ is negative at 1% significance. The inflection point of digital economy moves to the left, which indicates that government makes digital economy slow down the role of industrial collaborative agglomeration and strengthens the impact of digital economy on industrial collaborative agglomeration.

**Table 8:** Moderating effect and mediation effect.

Variables	(1)	(2)	(3)	(4)
	COAGGLO	COAGGLO	CREAT	COAGGLO
DIGE	-0.331***	-0.158***	4.833***	-0.180***
	(-21.24)	(-11.15)	(3.703)	(-13.07)
DIGE^2^	1.053***	0.511***	-6.198**	0.529***
	(35.96)	(18.35)	(-2.336)	(19.69)
GM×DIGE	0.901***			
	(20.59)			
GM× DIGE^2^	-2.545***			
	(-31.54)			
EA				0.004***
				(14.65)
lnHUM	2.85e-08***	2.46e-08***	-9.65e-07***	2.52e-08***
	(6.666)	(5.464)	(-7.982)	(5.511)
IS	-0.00139***	0.00121***	-0.258***	0.00204***
	(-3.223)	(2.609)	(-7.858)	(4.504)
lnGDP	0.00743***	0.00306***	0.270***	0.000665
	(8.761)	(3.774)	(7.028)	(0.824)
URB	0.0513**	0.0723***	2.873***	0.0579**
	(2.197)	(3.089)	(5.205)	(2.432)
LM	0.0586***	0.0578***	5.062***	0.0362***
	(52.43)	(42.19)	(40.68)	(18.22)
Constant	0.0161	0.0103	-1.537***	0.0320***
	(1.501)	(1.164)	(-3.937)	(3.640)
Observations	3,135	3,135	3,135	3,135

Note

*, **, and *** indicate p<0.1, p<0.05, and p<0.01, respectively; numbers in parentheses are t-values.

To investigate hypothesis H3, this research introduces the variable of urban entrepreneurial activity to explore the indirect impact of digital economy on industrial collaborative agglomeration. From columns (2) to (4) in Table 8, it can be seen that the regression coefficients of digital economy are significant at 1% level, and the regression coefficients of the urban entrepreneurial activity (EA) are also significant at 1% level. This suggests that digital economy can promote industrial collaborative agglomeration by improving urban entrepreneurial activity.

## 4. Conclusion and discussion

This research analyzes the impact of digital economy on industrial collaborative agglomeration based on the panel data of 286 prefecture-level cities from 2011 to 2021 and has the following findings. First, digital economy has a significant positive impact on industrial collaborative agglomeration. This is consistent with the finding of Huang et al [70]. It suggests that digital economy has emerged as a new driving force for the development of industrial collaborative agglomeration due to its high penetration and strong diffusion ability. Although digital economy has a positive role in promoting industrial collaborative agglomeration, this study shows that the impact of digital economy on industrial collaborative agglomeration is a U-shaped relation. The exact location of ’inflection point’ has been identified through empirical methods, which provides a new perspective for understanding the relationship between digital economy and industrial collaborative agglomeration. This finding implies that the connection of digital economy and industrial collaborative agglomeration is a long and complex process. Second, the results of this study confirm the role of entrepreneurial activity in bridging the gap between digital economy development and industrial collaborative agglomeration. In addition, government intervention has a role in promoting the impact of digital economy on industrial collaborative agglomeration. It indicates that not only digital economy itself, but also entrepreneurial activities and government intervention affects industrial collaborative agglomeration. These findings in this research offer a new perspective on how digital economy impacts industrial collaborative agglomeration and provide a theoretical basis for policymakers to formulate relevant policies.

Based on above findings, the following recommendations are proposed. First, it is necessary to fully utilize the multiplier effects of digital economy, by breaking through the boundaries of geography, organization and technology. This can promote the optimization of resource allocation from single-industry to multi-industry optimization, which in turn helps industrial collaborative agglomeration. Second, close attention should be paid to the non-linear impact of digital economy on industrial collaborative agglomeration. In line with the concept of sustainable development, policy makers need consider the impact of digital economy on industrial collaborative agglomeration and make appropriate adjustments to ensure the stable role of digital economy. Thirdly, it is crucial to promote inter-regional cooperation in China. To achieve collaborative development, it is important for all regions to cooperate and promote digital economy, considering the significant differences in its development across regions. Fourth, government intervention should be rationalized. Policies should be formulated to support digital technology from the perspective of industrial collaborative agglomeration and development. Investment in the construction of new digital infrastructure, such as the Internet, big data, and artificial intelligence, should be increased to ensure that digital technology can be fully applied and promoted in the process of industrial collaborative agglomeration. Finally, in order to encourage residents to engage in innovation and entrepreneurship, government should increase its financial support, create a good environment for innovation and entrepreneurship, and enhance urban entrepreneurial activity.

The limitation of this research is that it primarily focuses on the impact of digital economy development on industrial collaborative agglomeration. It does not explore in depth whether there will be spatial spillover effects, such as whether the development of local digital economy impacts the industrial collaborative agglomeration in neighboring regions. Future studies can examine the spatial spillover effects of the digital economy and investigate whether digital economy promotes the industrial structure upgrading.
